# Stationary Anonymous Sequential Games with Undiscounted Rewards

**DOI:** 10.1007/s10957-014-0649-9

**Published:** 2014-09-09

**Authors:** Piotr Więcek, Eitan Altman

**Affiliations:** 1 Institute of Mathematics and Computer Science, Wrocław University of Technology, Wybrzeże Wyspiańskiego 27, 50-370 Wrocław, Poland; 2 INRIA, 2004 Route des Lucioles, P.B. 93, 06902 Sophia Antipolis Cedex, France

**Keywords:** Stochastic game, Population game, Anonymous sequential game, Average reward, Total reward, Stationary policy, 91A15, 91A13, 91A25

## Abstract

Stationary anonymous sequential games with undiscounted rewards are a special class of games that combine features from both population games (infinitely many players) with stochastic games. We extend the theory for these games to the cases of total expected reward as well as to the expected average reward. We show that in the anonymous sequential game equilibria correspond to the limits of those of related finite population games as the number of players grows to infinity. We provide examples to illustrate our results.

## Introduction

Games with a continuum of atomless (or infinitesimal) players have since long ago been used to model interactions involving a large number of players, in which the action of a single player has a negligible impact on the utilities of other players. In road traffic engineering, for example, this was already formalized by Wardrop [1] in 1952 to model the choice of routes of cars where each driver, modeled as an atomless player, minimizes its expected travel delay. In Wardrop’s model, there may be several classes of players, each corresponding to another origin-destination pair. The goal is to determine, what fraction of each class of players would use the different possible paths available to that class. The equilibrium is known to behave as the limit of the equilibria obtained in games with finitely many players, as their number tends to infinity [2]. It is also the limit of Nash equilibria for some sequence of dynamic games in which randomness tends to average away as the number of players increases [3].

Another class of games that involves a continuum of atomless players is evolutionary games, in which pairs of players that play a matrix game are selected at random, see [4]. Our objective is again to predict the fraction of the population (or of populations in the case of several classes) that plays each possible action at equilibrium. A Wardrop type definition of equilibrium can be used, although there has been a particular interest in a more robust notion of equilibrium strategy, called Evolutionary Stable Strategy (we refer the reader to [5, 6]).

In both games described above, the player’s type is fixed, and the actions of the players determine directly their utilities.

Extensions of these models are needed, whenever the player’s class may change randomly in time, and when the utility of a player depends not only on the current actions of players but also on future interactions. The class of the player is called its individual state. The choice of an action by a player should then take into account not only the game played at the present state but also the future state evolution. We are interested in particular in the case where the action of a player not only impacts the current utility but also the transition probabilities to the next state.

In this paper, we study this type of extension in the framework of the first type of game, in which a player interacts with an infinite number of other players. (In the road traffic context, the interaction is modeled through link delays, each of which depends on the total amount of traffic that uses that link.) We build upon the framework of anonymous sequential games, introduced by Jovanovic and Rosenthal in 1988 in [7]. In that work, each player’s utility is given as the expected discounted utility over an infinite horizon. The theory of anonymous sequential games with discounted utilities was further developed in [8–12]. Conditions under which Nash equilibria in finite-player discounted-utility games converge to equilibria of respective anonymous models were analyzed in [13–16]. Also applications of this kind of models were numerous: from stochastic growth [17] and industry dynamics [18–21] models to dynamic auctions [22–24] and strategic market games [25, 26]. Surprisingly, the cases of expected average utility and total expected utility have remained open ever since 1988, even though this kind of models were applied in some networking contexts [27, 28]. Our main contribution in this paper is giving conditions, under which such extensions are possible.

Similar extensions have been proposed and studied for the framework of evolutionary games in [29, 30]. The analysis there turns out to be simpler, since the utility in each encounter between two players turns out to be bilinear there.

The structure of the paper is as follows. We begin with a section that presents the model and introduces in particular the expected average and the total expected reward criteria. The two following sections establish the existence of stationary equilibria for the average and the total reward (Sects. 3 and 4, respectively). Section 5 is concerned with showing that the equilibria for models of the two previous chapters that deal with infinite number of players are limits of those obtained for some games with a large finite number of players, as this number goes to infinity. We end with two sections that show how our results apply to some real-life examples, followed by two paragraphs, containing open problems and conclusions.

## The Model

The anonymous sequential game is described by the following objects:

- We assume that the game is played in discrete time that is $t\in \{ 1,2,\ldots \}$.
- The game is played by an infinite number (continuum) of players. Each player has his own private state $s\in S$, changing over time. We assume that $S$ is a finite set.
- The global state, $\mu ^t$, of the system at time $t$, is a probability distribution over $S$. It describes the proportion of the population, which is at time $t$ in each of the individual states. We assume that each player has an ability to observe the global state of the game, so from his point of view the state of the game at time $t$ is^1^ $(s_t,\mu ^t)\in S\times \Delta (S)$.
- The set of actions available to a player in state $(s,\mu )$ is a nonempty set $A(s,\mu )$, with $A:=\bigcup _{(s,\mu )\in S\times \Delta (S)}A(s,\mu )$—a finite set. We assume that the mapping $A$ is an upper semicontinuous function.
- Global distribution of the state-action pairs at any time $t$ is given by the measure $\tau ^t\in \Delta (S\times A)$. The global state of the system $\mu ^t$ is the marginal of $\tau ^t$ on $S$.
- An individual’s immediate reward at any stage $t$, when his private state is $s_t$, he plays action $a_t$, and the global state-action measure is $\tau ^t$ is $u(s_t,a_t,\tau ^t)$. It is a (jointly) continuous function.
- The transitions are defined for each individual separately with the transition function $Q:S\times A\times \Delta (S\times A)\rightarrow \Delta (S)$, which is also a (jointly) continuous function. We will write $Q(\cdot |s_t,a_t,\tau ^t)$ for the distribution of the individual state at time $t+1$, given his state at time $t$, $s_t$, his action $a_t$, and the state-action distribution of all the players.
- The global state at time $t+1$ will be given by^2^
  $$\begin{aligned} \Phi (\cdot |\tau ^t)=\sum _{s\in S}\sum _{a\in A}Q(\cdot |s,a,\tau ^t)\tau ^t_{sa}. \end{aligned}$$

Any function $f:S\times \Delta (S)\rightarrow \Delta (A)$ satisfying $\text{ supp }f(s,\mu )\subset A(s,\mu )$ for every $s\in S$ and $\mu \in \Delta (S)$ is called a *stationary policy*. We denote the set of stationary policies in our game by $\mathcal {U}$.

### Average Reward

We define the *long-time average reward* of a player using stationary policy $f$ when all the other players use policy $g$, and the initial state distribution (both of the player and his opponents) is $\mu ^1$, to be

$$\begin{aligned} J(\mu ^1,f,g)=\limsup _{T\rightarrow \infty }\frac{1}{T}E^{\mu ^1,Q,f,g}\sum _{t=1}^Tu(s_t,a_t,\tau ^t). \end{aligned}$$

Further, we define a stationary strategy $f$ and a measure $\mu \in \Delta (S)$ to be an equilibrium in the long-time average reward game iff for every other stationary strategy $g\in \mathcal {U}$,

$$\begin{aligned} J(\mu ,f,f)\ge J(\mu ,g,f) \end{aligned}$$

and, if $\mu ^1=\mu$, and all the players use policy $f$ then $\mu ^t=\mu$ for every $t\ge 1$.

#### *Remark 2.1*

The definition of the equilibrium used here differs significantly from that used in [7]. There the stationary equilibrium is defined as a state-action distribution $\tau$ with $\tau _s=\mu$, such that the Bellman equation for a player maximizing his discounted reward against others playing according to $\tau$ is satisfied $\tau$-a.s. Our definition directly relates it to the reward functionals.

### Total Reward

To define the total reward in our game, let us distinguish one state in $S$, say $s_0$, and assume that $A(s_0,\mu )=\{ a_0\}$ independently of $\mu$ for some fixed $a_0$. Then the *total reward* of a player using stationary policy $f$ when all the other players apply policy $g$, and the initial distribution of the states of his opponents is $\mu ^1$, while his own is $\rho ^1$, is defined in the following way:

$$\begin{aligned} \overline{J}(\rho ^1,\mu ^1,f,g)=E^{\rho ^1,\mu ^1,Q,f,g}\sum _{t=1}^{\mathcal {T}-1}u(s_t,a_t,\tau ^t), \end{aligned}$$

where $\mathcal {T}$ is the moment of the first arrival of the process $s_t$ to $s_0$. We interpret it as the reward accumulated by the player over whole of his lifetime. State $s_0$ is an artificial state (so is action $a_0$), denoting that a player is dead. $\mu ^1$ is the distribution of the states across the population when he is born, while $\rho ^1$ is the distribution of initial states of new-born players. The fact that after some time the state of a player can become again different from $s_0$ should be interpreted as that after some time the player is replaced by some new-born one.

The notion of equilibrium for the total reward case will be slightly different from that for the average reward. We define a stationary strategy $f$ and a measure $\mu \in \Delta (S)$ to be in equilibrium in the total reward game iff for every other stationary strategy $g\in \mathcal {U}$,

$$\begin{aligned} \overline{J}(\rho ,\mu ,f,f)\ge \overline{J}(\rho ,\mu ,g,f), \end{aligned}$$

where $\rho =Q(\cdot |s_0,a_0,\tau (f,\mu ))$ and $(\tau (f,\mu ))_{sa}=\mu _s(f(s))_a$ for all $s\in S$, $a\in A$, and, if $\mu ^1=\mu$, and all the players use policy $f$, then $\mu ^t=\mu$ for every $t\ge 1$.

## Existence of the Stationary Equilibrium in Average-Reward Case

In the present section, we present a result about the existence of stationary equilibrium in anonymous sequential games with long-time average reward. We prove it under the following assumption:

**(A1)** The set of individual states of any player $S$ can be partitioned into two sets $S_0$ and $S_1$ such that for every state-action distribution of all the other players $\tau \in \Delta (S\times A)$:

- All the states from $S_0$ are transient in the Markov chain of individual states of a player using any $f\in \mathcal {U}$.
- The set $S_1$ is strongly communicating.

There are a couple of equivalent definitions of “strongly communicating” property used above appearing in the literature. We follow the one formulated in [31], saying that a set $S_1$ of states in a Markov Decision Process is strongly communicating iff there exists a stationary policy^3^ $\overline{f}^\tau$ such that the probability of going from any state $s\in S_1$ to any other $s'\in S_1$ for a player using $\overline{f}^\tau$ is positive.

Assumption (A1) appears often in the literature on Markov decision processes with average cost and is referred to as “weakly communicating” property, see e.g., [32], chapters 8 and 9.^4^ It guarantees that the optimal gain in a Markov decision process satisfying it is independent of its initial state. As we will see, it also guarantees that this optimal gain is continuous in $\tau$, which will be crucial in proving the existence of an equilibrium in our game. It is also worth noting that without assumption (A1) the average-reward anonymous sequential game may have no stationary equilibria at all. This is shown in the following example^5^

### *Example 3.1*

Let us consider an average-reward anonymous sequential game with $S=\{ 1,2,3\}$, and

$$\begin{aligned} A(s,\mu )=\left\{ \begin{array}{ll} \{ 0,1\},&{}\quad \text{ if } s=1,\\ \{ 0\},&{}\quad \text{ otherwise },\end{array}\right. \end{aligned}$$

thus, the decision is only made by players in state $1$. For the simplicity we will denote this only decision by $a$ in what will follow. The immediate rewards for the players depend only on their private state as follows: $u(s)=3-s$. Finally, the transition matrix of the Markov chain of private states of each player is

$$\begin{aligned} \mathbb {Q}(a,\tau )=\left[ \begin{array}{lll} 1-\frac{a+3p^*}{4}&{}\frac{a}{4}&{}\frac{3p^*}{4}\\ \frac{1}{2}&{}\frac{1}{2}&{}0\\ \frac{p^*}{2}&{}0&{}1-\frac{p^*}{2} \end{array}\right] , \text{ where } p^*=\max \{ 0,1-4\tau _{11}\}. \end{aligned}$$

It violates assumption (A1), as e.g., when $\tau _{11}\ge \frac{1}{4}$ for the pure strategy assigning $a=0$ in state $1$, states $1$ and $2$ are absorbing in the Markov chain of individual states of a player, and state $2$ is transient, while when it assigns $a=1$, states $1$ and $2$ become communicating. We will show that such a game has no stationary equilibrium.

Suppose that $(f,\mu ^*)$ is an equilibrium. We will consider two cases:

- $\tau _{11}\ge \frac{1}{4}$ for $\tau$ corresponding to $\mu ^*$ and $f$. Then $p^*=0$, and so if a player uses action $1$ with probability $\beta$, the stationary state of the chain of his states when his initial state’s distribution is $\mu ^*$ is $\left[ \frac{2(\mu ^*_1+\mu ^*_2)}{2+\beta },\frac{\beta (\mu ^*_1+\mu ^*_2)}{2+\beta },\mu ^*_3\right]$, and his long-time average reward is
  $$\begin{aligned} \frac{(4+\beta )(\mu ^*_1+\mu ^*_2)}{2+\beta }=\left( 1+\frac{2}{2+\beta }\right) (\mu ^*_1+\mu ^*_2), \end{aligned}$$
  which is a strictly decreasing function of $\beta$ (recall that $\mu ^*_1\ge \tau _{11}\ge \frac{1}{4}$). Thus his best response to $f$ is the policy, which assigns probability $1$ to action $a=0$ in state $1$. But if all the players use such policy, $\tau _{11}=0$, which contradicts our assumption that it is no less than $\frac{1}{4}$.
- $\tau _{11}<\frac{1}{4}$. Then it can be easily seen that the stationary state of any player’s chain when he uses action $1$ with probability $\beta \in [0,1]$ is independent of the initial distribution of his state $\mu ^*$ and equal to $\left[ \frac{2}{5+\beta },\frac{\beta }{5+\beta },\frac{3}{5+\beta }\right]$, which gives him the reward of $\frac{4+\beta }{5+\beta }=1-\frac{1}{5+\beta }$, which is clearly a strictly increasing function of $\beta$. Thus the best response to $f$ is to play action $a=1$ with probability $1$, which, if applied by all the players, results in stationary state $\mu ^*=\left[ \frac{1}{3},\frac{1}{6},\frac{1}{2}\right]$ and consequently $\tau _{11}=\frac{1}{3}$, contradicting the assumption that it is less than $\frac{1}{4}$.

Thus this game cannot have a stationary equilibrium.

Now we are ready to formulate the main result of this section.

### **Theorem 3.1**

Every anonymous sequential game with long-time average payoff satisfying (A1) has a stationary equilibrium.

Before we prove the theorem, let us introduce some additional notation. We will consider a Markov decision process $\mathcal {M}(\tau )$ of an individual faced with a fixed (over time) distribution of state-action pairs of all the other players. For this fixed $\tau \in \Delta (S\times A)$, let $J^\tau (f,\mu )$ denote the long-time average payoff in this process when the player uses stationary policy $f$, and the initial distribution of states is $\mu$ that is

$$\begin{aligned} J^\tau (f,\mu )=\limsup _{T\rightarrow \infty }\frac{1}{T}E^{\mu ,Q,f}\sum _{t=1}^Tu(s_t,a_t,\tau ). \end{aligned}$$

By well-known results from dynamic programming (see e.g., [32]), in a weakly communicating Markov decision process (this is such a process by (A1)), the optimal gain is independent of $\mu$. We denote this uniform optimal gain by $G(\tau )$ that is

$$\begin{aligned} G(\tau )=\sup _{f:S\rightarrow A}J^\tau (f,\mu )\quad \text{ for } \text{ any } \text{ fixed } \mu \in \Delta (S). \end{aligned}$$

Lemma 3.2 states a crucial feature of $G$. It is preceded by another technical one.

### **Lemma 3.1**

Suppose that $\mu (n)$ are invariant measures of Markov chains with common finite state set $S$ and with transition matrices $P(n)$, respectively. Then if ${\mu (n)\rightarrow \mu }$ and $P(n)\rightarrow P$, then $\mu$ is an invariant measure for the Markov chain with transition matrix $P$.

### *Proof*

By the definition of invariant measure every $\mu (n)$ satisfies for every $s\in S$

$$\begin{aligned} (\mu (n))_s=\sum _{i\in S}(\mu (n))_i(P(n))_{si}. \end{aligned}$$

If we pass to the limit, we obtain

$$\begin{aligned} \mu _s=\sum _{i\in S}\mu _iP_{si}, \end{aligned}$$

which means that $\mu$ is an invariant measure for the Markov chain with transition matrix $P$. $\square$

### **Lemma 3.2**

Under (A1), $G$ is a continuous function of $\tau$.

### *Proof*

Let $\tau ^n$ be a sequence of probability measures on $S\times A$ converging to $\tau$. Since all of the MDPs we consider here have finite state space, each of them has a stationary optimal policy^6^, say policy $f^n$ is optimal in $\mathcal {M}(\tau ^n)$. Next, let $\mu ^n$ be an invariant measure corresponding to strategy $f^n$ in $\mathcal {M}(\tau ^n)$ (by (A1) such a measure exists, maybe more than one). Such an invariant measure must satisfy

1 $$\begin{aligned} G(\tau ^n)=\sum _{s\in S}\sum _{a\in A}(f^n(s))_a\mu ^n_su(s,a,\tau ^n), \end{aligned}$$

otherwise $f^n$ would not be optimal.

Note next that $\mathcal {U}$ and $\Delta (S)$ are compact sets. Thus, there exists a subsequence of $f^n$ converging to some $f^0\in \mathcal {U}$ and then a subsequence of $\mu ^n$ converging to some $\mu ^0$. Without a loss of generality, we may assume that these are sequences $f^n$ and $\mu ^n$ that are convergent. By the continuity assumption about $Q$ and Lemma 3.1, $\mu ^0$ is an invariant measure corresponding to $f^0$ in $\mathcal {M}(\tau )$. If we next pass to the limit in (1), we get

$$\begin{aligned} \lim _{n\rightarrow \infty }G(\tau ^n)=\sum _{s\in S}\sum _{a\in A}(f^0(s))_a\mu ^0_su(s,a,\tau )=J^\tau (f^0,\mu ^0). \end{aligned}$$

But this implies that $G(\tau )\ge \lim _{n\rightarrow \infty }G(\tau ^n)$.

To show the inverse inequality, suppose that $f\in \mathcal {U}$ is an optimal policy in $\mathcal {M}(\tau )$. By (A1) the states in the Markov chain of individual states for a user applying $f$ in $\mathcal {M}(\tau )$ can be divided into a class of transient states and a number of communicating classes. Let $S^*$ be a communicating class such that the ergodic payoff in this class is equal to $G(\tau )$. Define now the policies $g^n$ in the following way:

$$\begin{aligned} g^n(s)=\left\{ \begin{array}{ll}f(s),&{}\text{ when } s\in S^*,\\ f^{\tau ^n}(s),&{}\text{ when } s\in S\setminus S^*.\end{array}\right. \end{aligned}$$

(Here $f^{\tau ^n}$ is a communicating policy derived from assumption (A1)). One can easily notice that under these policies applied in $\mathcal {M}(\tau ^n)$, all the states from $S\setminus S^*$ would be transient. Now let $\overline{\mu }^n$ be an invariant measure corresponding to $g^n$ in $\mathcal {M}(\tau ^n)$. Again using Lemma 3.1 we can show that the limit (possibly over a subsequence) of the sequence $\overline{\mu }^n$, say $\overline{\mu }^0$ is an invariant measure of the limit of $g^n$, which is equal to $f$ on $S^*$. At the same time, $\overline{\mu }^0_s=0$ for $s\in S\setminus S^*$, so we can write (from the definition of invariant measure) for every $s\in S^*$:

$$\begin{aligned} \overline{\mu }^0_s&= \sum _{i\in S}\sum _{a\in A}Q(s|i,a,\tau )(g^0(i))_a\overline{\mu }^0_i=\sum _{i\in S^*}\sum _{a\in A}Q(s|i,a,\tau )(g^0(i))_a\overline{\mu }^0_i\\&= \sum _{i\in S^*}\sum _{a\in A}Q(s|i,a,\tau )(f(i))_a\overline{\mu }^0_i=\sum _{i\in S}\sum _{a\in A}Q(s|i,a,\tau )(f(i))_a\overline{\mu }^0_i, \end{aligned}$$

which means that $\overline{\mu }^0$ is also an invariant measure for $f$, and it is entirely concentrated on $S^*$. But since $S^*$ is a communicating class under $f$, this implies

$$\begin{aligned} J^\tau (g^0,\overline{\mu }^0)=J^\tau (f,\overline{\mu }^0)=G(\tau ). \end{aligned}$$

On the other hand

$$\begin{aligned} J^\tau (g^0,\overline{\mu }^0)&= \sum _{s\in S}\sum _{a\in A}(g^0(s))_a\overline{\mu }^0_su(s,a,\tau )\\&= \lim _{n\rightarrow \infty }\sum _{s\in S}\sum _{a\in A}(g^n(s))_a\overline{\mu }^n_su(s,a,\tau ^n)\\&= \lim _{n\rightarrow \infty }J^{\tau ^n}(g^n,\overline{\mu }^n)\le \lim _{n\rightarrow \infty }G(\tau ^n), \end{aligned}$$

ending the proof. $\square$

### *Proof of Theorem3.1*

We consider two multifunctions of $\tau \in \Delta (S\times A)$:

2 $$\begin{aligned} B(\tau ):=\left\{ \rho \in \Delta (S\times A): \sum _{s\in S}\sum _{a\in A}\rho _{sa}u(s,a,\tau )=G(\tau )\right\} ,\end{aligned}$$

3 $$\begin{aligned} C(\tau ):=\left\{ \rho \in \Delta (S\times A):\sum _{a\in A}\rho _{sa}=\sum _{x\in S}\sum _{b\in B}Q(s|x,b,\tau )\rho _{xb}\right\} , \end{aligned}$$

and let $\Psi (\tau ):=B(\tau )\cap C(\tau )$. We will show that $\Psi$ has a fixed point, and then that this fixed point corresponds to an equilibrium in the game.

First, note that $C(\tau )$ is the set of all the possible stationary state-action measures in $\mathcal {M}(\tau )$. By Theorem 1 in [35] it is also the set of occupation measures corresponding to all the possible stationary policies and all the possible initial distributions of states in $\mathcal {M}(\tau )$. Since $G(\tau )$ is the optimal reward in this MDP, there exists a stationary policy and thus an occupation measure corresponding to it, for which the reward is equal to $G(\tau )$. This implies that for any $\tau$, $\Psi (\tau )$ is nonempty. Further, note that for any $\tau$, both $B(\tau )$ and $C(\tau )$ are trivially convex and thus so is their intersection. Finally, as an immediate consequence of Lemma 3.1, $C$ has a closed graph. On the other hand, the closedness of the graph of $B$ is a trivial consequence of the continuity of $u$ (by assumption) and $G$ (by Lemma 3.2). The graph of $\Psi$ is the intersection of the two and thus is also closed. The existence of a fixed point of $\Psi$ follows now from Glickberg’s fixed point theorem [36].

Now suppose $\tau ^*$ is this fixed point. Since it is a fixed point of $C$, it satisfies

$$\begin{aligned} \sum _{a\in A}\tau ^*_{sa}=\sum _{x\in S}\sum _{b\in B}Q(s|x,b,\tau ^*)\tau ^*_{xb}=\Phi (s|\tau ^*). \end{aligned}$$

This implies that if the initial distribution of states is $\tau ^*_S$, and players apply stationary policy $f$ defined for any $\tau \in \Delta (S\times A)$ by^7^

4 $$\begin{aligned} (f(s),\tau )_a=\left\{ \begin{array}{ll}\frac{\tau ^*_{sa}}{\sum _{b\in A}\tau ^*_{sa}},&{}\quad \text{ if } \sum _{b\in A}\tau ^*_{sa}>0,\\ \delta [a_0],&{}\quad \text{ otherwise, }\end{array}\right. \end{aligned}$$

for any fixed $a_0\in A$, the distribution of state-action pairs in the population is always $\tau ^*$. On the other hand, since $\tau ^*\in B(\tau ^*)$, $f$ is the best response of a player when the state-action distribution is always $\tau ^*$ and thus together with $\tau ^*_S$ an equilibrium in the game. $\square$

## Existence of the Stationary Equilibrium in Total Reward Case

In this section, we show that also for the total reward case under some fairly mild assumptions, the game has an equilibrium. What we will assume is the following:

**(T1)**: There exists a $p_0>0$ such that for any fixed state-action measure $\tau$, and under any stationary policy $f$, the probability of getting from any state $s\in S\setminus \{ s_0\}$ to $s_0$ in $|S|-1$ steps is not smaller than $p_0$.

We write that this assumption is fairly mild, as it is not only necessary for our theorem to hold but also for the total cost model to make sense, as it is trivially shown in an example below.

### *Example 4.1*

Consider an anonymous sequential game with total cost with ${S=\{ 1,2,3\}}$ (and state $s_0$ denoted here as $s=0$) and

$$\begin{aligned} A(s,\mu )=\left\{ \begin{array}{ll} \{ 1,2\},&{}\quad \text{ if } s=1,\\ \{ 1\},&{}\quad \text{ otherwise }.\end{array}\right. \end{aligned}$$

The immediate rewards for the players depend only on their private state as follows:

$$\begin{aligned} u(s)=\left\{ \begin{array}{ll}1,&{}\quad \text{ if } s=1,\\ -1,&{}\quad \text{ otherwise }.\end{array}\right. \end{aligned}$$

Finally, the transitions of the Markov chain of private states of each player are defined as follows: if his action in state $1$ is $1$, he moves with probability $1$ to state $2$; if his action is $2$, he moves with probability $1$ to state $3$. In state $2$ he moves to $1$ with probability $1$, while in state $3$ he dies with probability $\frac{1}{2}$ and stays in $3$ with probability $\frac{1}{2}$. This game is completely decoupled (in the sense that neither the rewards nor the transitions of any player depend on those of the others), so it is easy to analyze.

It is immediate to see that under pure stationary policy choosing action $1$ in state $1$, a player never dies. Moreover, he receives payoffs of $1$ and $-1$ in subsequent periods, so his reward (the sum of these rewards over his lifetime) is not well defined. If we try to correct it by defining the total reward as the $\limsup$ or $\liminf$ of his accumulated rewards after $n$ periods of his life, we obtain a strange situation that the policy choosing action $1$ is optimal for each of the players in the $\limsup$ version of the reward and his worst possible policy for the $\liminf$ version.

### *Remark 4.1*

The total reward model, specifically when (T1) is assumed, bears a lot of resemblance to an exponentially discounted model where the discount factor is allowed to fluctuate over time, which suggests that the results in the two models should not differ much. Note, however, that there is one essential difference between these two models. The ‘discount factor’ in the total reward model (which is the ratio of those who stay alive after a given period to those who were alive at its beginning) appears not only in the cumulative reward of the players but also in the stationary state of the game, and thus also in the per-period rewards of the players. Thus this is an essentially different (and slightly more complex) problem. On the other hand, the fact that each of the players lives for a finite period and then is replaced by another player, with a fixed fraction of players dead and fixed fractions of players in each of the states when the game is in a stationary state, makes this model similar to the average reward one. In fact, using the renewal theorem, we can relate the rewards of the players in the total reward model with those in the respective average-reward model. This relation is used a couple of times in our proofs.

Now we can formulate our main result of this section.

### **Theorem 4.1**

Every anonymous sequential game with total reward satisfying (T1) has a stationary equilibrium.

As in the case of the average reward, we start by defining some additional notation. Let $\overline{\mathcal {M}}(\tau )$ be a modified Markov decision process of an individual faced with a fixed (over time) distribution of state-action pairs of all the other players. This modification is slight but important, namely we assume that in $\overline{\mathcal {M}}(\tau )$ the state $s_0$ is absorbing, and the reward in this state is always 0. This is a classic MDP with total reward, as considered in the literature. For this fixed $\tau \in \Delta (S\times A)$, let $\overline{J}^\tau (f,\rho )$ denote the total payoff in this process when the player uses stationary policy $f$, and the initial distribution of states is $\rho$, and let $\overline{G}(\tau )$ denote the optimal reward in $\overline{\mathcal {M}}(\tau )$ that is

$$\begin{aligned} \overline{G}(\tau )=\sup _{f:S\rightarrow A}\overline{J}^\tau (f,Q(\cdot |s_0,a_0,\tau )). \end{aligned}$$

We can prove the following auxiliary result:

### **Lemma 4.1**

Under (T1), $\overline{J}^\tau (f,\rho )$ is a (jointly) continuous function of $\rho$, $f$, and $\tau$.

### *Proof*

By a well-known result from the theory of Markov decision processes (see e.g., [32], Lemma 7.1.8 – assumption of this lemma is satisfied as a consequence of assumption (T1) and the fact that function $u$ is continuous on a compact set and thus bounded), $\overline{J}^\tau (f,\rho )$ is the limit of the rewards in respective discounted MDPs $\overline{J}_\beta ^\tau (f,\rho )$ as the discount factor $\beta$ approaches 1. Since by a well-known result (see e.g., Lemma 8.5 in [37]), discounted rewards are continuous functions of stationary policies, and they are linear in the initial distribution of states, to show the continuity of $\overline{J}^\tau (f,\rho )$, it is enough to prove that the convergence of $\overline{J}_\beta ^\tau (f,\rho )$ is uniform.

Let us take an $\varepsilon >0$ and set $M=\max _{s,a,\tau }|u(s,a,\tau )|$ (such a number exists, as $u$ is a continuous function defined on a compact set). The probability that after $m(|S|-1)$ steps the state of an individual did not reach $s_0$ is for any stationary policy $f$ by assumption (T1) not greater than $(1-p_0)^m$. This means that for any $m$,

5 $$\begin{aligned} \left| \overline{J}^\tau (f,\rho )-\sum _{t=1}^{m(|S|-1)}E^{\rho ,Q,f}u(s_t,a_t,\tau )\right| \le M(|S|-1)(1-p_0)^m, \end{aligned}$$

which for $m$ big enough, say bigger than $m_\varepsilon$, is not greater than $\frac{\varepsilon }{3}$. Note that analogously for $m\ge m_\varepsilon$ and any $\beta \in (0,1)$

6 $$\begin{aligned} \left| \overline{J}_\beta ^\tau (f,\rho )-\sum _{t=1}^{m(|S|-1)}E^{\rho ,Q,f}\beta ^{t-1}u(s_t,a_t,\tau )\right| <\frac{\varepsilon }{3}. \end{aligned}$$

Next, note that

7 $$\begin{aligned}&\left| \sum _{t=1}^{m(|S|-1)}E^{\rho ,Q,f}u(s_t,a_t,\tau )-\sum _{t=1}^{m(|S|-1)}E^{\rho ,Q,f}\beta ^{t-1}u(s_t,a_t,\tau )\right| \nonumber \\&\quad \le M(1-\beta ^{m(|S|-1)})\le \frac{\varepsilon }{3} \end{aligned}$$

for $\beta$ big enough, say $\beta >\beta _\varepsilon$. Combining (5), (6), and (7) and using the triangle inequality, we obtain

$$\begin{aligned}&\left| \overline{J}^\tau (f,\rho )-\overline{J}_\beta ^\tau (f,\rho )\right| \le \left| \overline{J}^\tau (f,\rho )-\sum _{t=1}^{m(|S|-1)}E^{\rho ,Q,f}u(s_t,a_t,\tau )\right| \\&\quad +\left| \sum _{t=1}^{m(|S|-1)}E^{\rho ,Q,f}u(s_t,a_t,\tau )-\sum _{t=1}^{m(|S|-1)}E^{\rho ,Q,f}\beta ^{t-1}u(s_t,a_t,\tau )\right| \\&\quad +\left| \sum _{t=1}^{m(|S|-1)}E^{\rho ,Q,f}\beta ^{t-1}u(s_t,a_t,\tau )-\overline{J}_\beta ^\tau (f,\rho )\right| \le \frac{\varepsilon }{3}+\frac{\varepsilon }{3}+\frac{\varepsilon }{3}=\varepsilon \end{aligned}$$

for $\beta >\beta _\varepsilon$, which ends the proof. $\square$

### *Proof of Theorem 4.1*

We shall consider two multifunctions of $\tau \in \Delta (S\times A)$:

$$\begin{aligned} \overline{B}(\tau ):&= \left\{ \rho \in \Delta (S\times A): \exists f^\rho \in \mathcal {U}\right. \\&\forall s\in S,\,\left( \sum _{a\in A}\rho _{sa}>0\Rightarrow f^\rho (s)=\frac{\rho _{sa}}{\sum _{a\in A}\rho _{sa}}\right) \\&\left. \text{ and } \overline{J}^\tau (f^\rho ,Q(\cdot |s_0,a_0,\tau ))=\overline{G}(\tau )\right\} , \end{aligned}$$

$$\begin{aligned} \overline{C}(\tau ):=\left\{ \rho \in \Delta (S\times A):\sum _{a\in A}\rho _{sa}=\sum _{x\in S}\sum _{b\in B}Q(s|x,b,\tau )\rho _{xb}\right\} , \end{aligned}$$

and let $\overline{\Psi }(\tau ):=\overline{B}(\tau )\cap \overline{C}(\tau )$. We will show that $\overline{\Psi }$ has a fixed point and that it corresponds to an equilibrium in the game.

First, note that $\overline{\Psi }(\tau )$ is nonempty for any $\tau \in \Delta (S\times A)$, as any invariant measure corresponding to an optimal stationary policy in $\overline{\mathcal {M}}(\tau )$ belongs to $\overline{\Psi }(\tau )$ (such an optimal stationary policy exists according to Theorem 7.1.9 in [32]). Next, we show that $\overline{\Psi }(\tau )$ is convex for every $\tau \in \Delta (S\times A)$. By the renewal theorem (Theorem 3.3.4 in [38]) the total reward occupation measure corresponding to some stationary policy $f$ is equal to the average-reward occupation measure under the same policy multiplied by the expected lifetime. This implies that for any $\rho \in \overline{\Psi }(\tau )$ (the notation used here is the same as in the definition of $\overline{B}(\tau )$)

8 $$\begin{aligned} \overline{G}(\tau )=\overline{J}^\tau (f^\rho ,Q(\cdot |s_0,a_0,\tau ))=E^{Q,f}\mathcal {T}\sum _{s\in S}\sum _{a\in A}\rho _{sa}u(s,a,\tau ). \end{aligned}$$

Next, note that the time spent in state $s_0$, playing action $a_0$ is independent of the policy used by the player. We shall denote this time by $\mathcal {T}_0$. Again by the renewal theorem we can write

$$\begin{aligned} E^{Q}\mathcal {T}_0=E^{Q,f}\mathcal {T}\frac{\rho _{s_0a_0}}{\sum _{s\in S}\sum _{a\in A}\rho _{sa}}=E^{Q,f}\mathcal {T}\rho _{s_0a_0}. \end{aligned}$$

Substituting this into (8) we obtain

$$\begin{aligned} \overline{G}(\tau )=\frac{E^{Q}\mathcal {T}_0}{\rho _{s_0a_0}}\sum _{s\in S}\sum _{a\in A}\rho _{sa}u(s,a,\tau ) \end{aligned}$$

or equivalently

$$\begin{aligned} E^Q\mathcal {T}_0\sum _{s\in S}\sum _{a\in A}\rho _{sa}u(s,a,\tau )-\overline{G}(\tau )\rho _{s_0a_0}=0. \end{aligned}$$

The set of probability measures $\rho$ satisfying the above equality is clearly a polytope, and hence a convex set. What we are left to show is that the graph of $\overline{\Psi }$ is closed. Suppose that $\tau _n,\tau ,\rho _n,\rho \in \Delta (S\times A)$, $\tau _n\rightarrow \tau$, $\rho _n\rightarrow \rho$ and $\rho _n\in \overline{\Psi }(\tau _n)$ for every $n$. By Lemma 3.1 $\rho \in \overline{C}(\tau )$. Clearly, as $\rho _n\rightarrow \rho$, also $f^{\rho _n}\rightarrow f^\rho$. Since $\rho _n\in \overline{B}(\tau _n)$,

$$\begin{aligned} \overline{G}(\tau _n)=\overline{J}^{\tau _n}(f^{\rho _n},Q(\cdot |s_o,a_0,\tau _n))\ge \overline{J}^{\tau _n}(g,Q(\cdot |s_o,a_0,\tau _n)) \end{aligned}$$

for any stationary strategy $g$. By Lemma 4.1 and the continuity of $Q$ also

$$\begin{aligned} \overline{J}^{\tau }(f^{\rho },Q(\cdot |s_o,a_0,\tau ))\ge \overline{J}^{\tau }(g,Q(\cdot |s_o,a_0,\tau )), \end{aligned}$$

implying that $\rho \in \overline{B}(\tau )$ and hence also in $\overline{\Psi }(\tau )$. This means that all the assumptions of the Glicksberg theorem [36] are satisfied, and $\overline{\Psi }$ has a fixed point.

Now suppose $\tau ^*$ is this fixed point. Because $\tau ^*\in \overline{C}(\tau ^*)$, it satisfies

$$\begin{aligned} \sum _{a\in A}\tau ^*_{sa}=\sum _{x\in S}\sum _{b\in B}Q(s|x,b,\tau ^*)\tau ^*_{xb}=\Phi (s|\tau ^*). \end{aligned}$$

On the other hand, notice that

$$\begin{aligned} \overline{J}(\tau ^*_S,g,f^{\tau ^*})=\overline{J}^{\tau ^*}(g,\rho ) \end{aligned}$$

for any stationary policy $g$ and any initial state distribution $\rho$, and so ${\tau ^*\in \overline{B}(\tau ^*)}$ implies that $f^{\tau ^*}$ is the best response of a player, when the state-action distribution of his opponents is always $\tau ^*$, and hence $(f^*,\tau ^*_S)$ with ${f^*(s,\tau )\equiv f^{\tau ^*}(s)}$ for any $\tau \in \Delta (S\times A)$ is an equilibrium in the game. $\square$

## The Relation with Games with Finitely Many Players

Main point of criticism of anonymous games in general is that the limiting situation with an infinite number of players does not exist in reality, and thus it is not sure that the results obtained for a continuum of players are relevant for the real-life ones, when the number of players is finite, but large. In this section, we present some results connecting the anonymous game models from previous sections with similar models with finitely many players. In these results we will, in addition, use the following two assumptions.

**(AT1)**: $Q(\cdot |a,s,\tau )=Q(\cdot |a,s)$ for all $\tau \in \Delta (S\times A)$ and $A(\cdot ,\mu )=A(\cdot )$ for all $\mu \in \Delta (S)$.
**(AT2)**: For any $f\in \mathcal {U}$ and $\tau \in \Delta (S\times A)$ the Markov chain of individual states of an individual using $f$, when the state-action distribution of all the other players is $\tau$, is aperiodic.

The assumptions similiar to (AT1) appear in a recent paper [39] on stochastic games with a finite number of players and average reward. They are also used in some papers on Markov evolutionary games [29] and in a recent application of anonymous sequential games to model power control problem in a wireless network [27]. It allows to decouple the Markov chains of individual states for each of the players (so that the dependence between different players is only through rewards)—this decoupling is crucial in our proofs of convergence of games with finite number of players to respective anonymous models. Importantly though, in most engineering applications individual state of a player is either his energy (or some other private resource) level or his geographical position for which assumption (AT1) is naturally satisfied.

We will need to define some additional notation.

- We say that an $n$-person stochastic game is the $n$-person counterpart of an anonymous game iff it is defined with the same objects $S$, $A$, $u$, and $Q$, with the difference that the number of players is $n$, and in consequence the global states and state-action distributions are defined on subsets of $\Delta (S)$ and $\Delta (S\times A)$,
  $$\begin{aligned}&\Delta ^n(S):=\{ \mu \in \Delta (S): \mu _s=\frac{k_s}{n}, k_s\in \mathbb {N}, \text{ for } \text{ all } s\in S\},\\&\Delta ^n(S\times A):=\{ \tau \in \Delta (S\times A): \sum _{a\in A}\tau _{sa}=\frac{k_s}{n}, k_s\in \mathbb {N}, \text{ for } \text{ all } s\in S\}. \end{aligned}$$
- We will consider a wider set of possible policies in the game. Namely, we will consider a situation, when each player uses a stationary policy over the whole game, but this policy is chosen at the beginning of the play according to some probability distribution. This means that any probability distribution from $\Delta (\mathcal {U})$ will be a policy in the game.
- We will consider a different (i.e., standard) definition of policies and equilibrium in the average-reward game. We will say that policies $(f^1,\ldots ,f^n)$ form a Nash equilibrium in the average-reward $n$-person game iff for any player $i$ and any initial distribution of the global state $\mu ^1$,
  $$\begin{aligned} J^i(\mu ^1,f^1,\ldots ,f^i,\ldots ,f^n)\ge J^i(\mu ^1,f^1,\ldots ,g^i,\ldots ,f^n) \end{aligned}$$
  for any other policy $g^i$. If this inequality holds up to some $\varepsilon$, we say that $(f^1,\ldots ,f^n)$ are in $\varepsilon$-equilibrium. For both models (with average and total reward) we will also consider the notion of equilibrium defined as for the anonymous game—we will call it then a *weak equilibrium* (and analogously define weak $\varepsilon$-equilibrium).

Now we can prove the following two results:

### **Theorem 5.1**

Suppose $(f,\mu )$ is an equilibrium in either an average-reward anonymous game satisfying (A1) and (AT1) or a total reward anonymous game satisfying (T1) and (AT1). Then for every $\varepsilon >0$ there exists an $\overline{n}_\varepsilon$ such that for every $n\ge \overline{n}_\varepsilon$ $(f,\mu )$ is a weak equilibrium in the $n$-person counterpart of this anonymous game.

A stronger result is true for the average-reward game.

### **Theorem 5.2**

For every $\varepsilon >0$ there exists an $n_\varepsilon$ such that for every $n\ge n_\varepsilon$ the $n$-person counterpart of the average-reward anonymous game satisfying (A1), (AT1) and (AT2) has a symmetric Nash equilibrium $(\pi ^n,\ldots ,\pi ^n)$, where $\pi ^n\in \Delta (\mathcal {U})$. Moreover if $(f,\mu )$ is an equilibrium in the anonymous game, then $\pi ^n$ is of the form:

$$\begin{aligned} \pi ^n(s)=\sum _{l}\mu ^*_l\delta [f^n_l(s)],\quad \text{ where }\quad f^n_l(s)=\left\{ \begin{array}{ll}\overline{f}(s),&{} \text{ if } s\not \in S_l,\\ f(s),&{} \text{ if } s\in S_l,\end{array}\right. \end{aligned}$$

$\overline{f}$ is the communicating policy induced by the assumption (A1)^8^, $S_l$ are ergodic classes of the individual state process of a player when he applies policy $f$, and $\mu ^*$ is the probability measure on these ergodic classes corresponding to measure $\mu$ over $S$ 9.

### *Proof of Theorem5.1*

Let $J^l(\mu ,g,f)$ denote the reward in the $n$-person counterpart of the given average-reward anonymous game for a player using stationary strategy $g$ against $f$ of all the others, when initial distribution of individual states is $\mu$.

$$\begin{aligned} J^n(\mu ,g,f)&= \lim _{T\rightarrow \infty }\frac{1}{T}E^{g,f,\mu ,Q}\sum _{t=1}^Tu(s_t,g(x_t),\tau ^t)\\&= E^{f,\mu ,Q}\sum _{x,a}\sigma _{xa}(g)m_f^n(\tau )u(x,a,\tau ), \end{aligned}$$

where $\sigma (g)$ is the occupation measure of the process of individual states of the player using policy $g$, and $m_f^n$ is a probability measure over the set $\Delta (S\times A)$ denoting the frequency of the appearance of different state-action measures of all the players over the course of the game. Note further that $\sigma (g)$ is under (AT1) independent of $n$ and the same as in the limiting (anonymous game) case. On the other hand, $m_f^n$ converges weakly as $n$ goes to infinity to the invariant measure corresponding to the policy $f$ and the initial distribution $\mu$. Thus,

$$\begin{aligned} J^n(\mu ,g,f)\rightarrow _{n\rightarrow \infty }J(\mu ,g,f) \end{aligned}$$

for any stationary strategy $g$. The thesis of the theorem follows immediately.

To prove the total reward case, we first need to write the reward in the $n$-person counterpart of the given total reward anonymous game for a player using stationary strategy $g$ against $f$ of all the others, when initial distribution of states is $\mu$:

$$\begin{aligned} \overline{J}^n(\mu ,g,f)=E^{g,f,\mu ,Q}\sum _{t=1}^\mathcal {T} u(s_t,g(x_t),\tau ^t), \end{aligned}$$

with $s_1$ distributed according to $Q(\cdot |s_0,a_0)$ (recall that by (AT1) this distribution is independent of the global state-action distribution $\tau$). This by the renewal theorem equals

$$\begin{aligned} E^{g,\mu ,Q}\mathcal {T}(g)\sum _{x,a}\sigma _{xa}(g)m_f^n(\tau )u(x,a,\tau ), \end{aligned}$$

where $\sigma (g)$ is the occupation measure of the process of individual states of the player using policy $g$, and $m_f^n$ is a probability measure over the set $\Delta (S\times A)$, denoting the frequency of the appearance of different state-action measures of all the players over the course of the game. But both $\sigma (g)$ and $E^{g,\mu ,Q}\mathcal {T}(g)$ are under (AT1) independent of $n$ and the same as in the limiting (anonymous game) case, while $m_f^n$ converges weakly to the invariant measure corresponding to the policy $f$ and the initial distribution $\mu$ as $n\rightarrow \infty$. The thesis of the theorem is now obtained as in the average-reward case. $\square$

### *Proof of Theorem5.2*

Fix $\varepsilon >0$ and take $n_\varepsilon$ such that for every $n\ge n_\varepsilon$ $(f,\ldots ,f)$ is a weak $\varepsilon$-equilibrium in the $n$-person counterpart of the average-reward game. Next, note that for every $n\in \mathbb {N}$ the process of individual states of each of the players using policy $\pi ^n$ has a unique invariant measure $\mu$. Since by (AT2) the process of individual states is aperiodic, it will converge to this invariant measure and, consequently, the reward for a player using policy $f$ against $f$ of all the others will be equal to $J(\mu ,f,f)$ for any initial state distribution. However, since in a weakly communicating Markov decision process the optimal gain is independent of this distribution, and since $(f,\ldots ,f)$ is a weak $\varepsilon$-equilibrium in the $n$-person game for $n\ge n_\varepsilon$, it is also a Nash $\varepsilon$-equilibrium in the $n$-person game. $\square$

## Application: Medium Access Game

In the remainder of the paper, we present two simple examples of application of our framework to model some real-life phenomena.

### The Model

The first example we present is a medium access game (MAC) between mobile phones. We are inspired by a power control game studied in [30] that considered a Markov Decision Evolutionary Game (MDEG) framework, which can be viewed as a special case of an anonymous sequential game (ASG), where the level of the power used for transmission is controlled. The total energy available for transmission is assumed to be constrained by the battery energy level, and the objective of a user is to maximize the amount of successful transmissions of packets taking into account the fact that the lifetime of its battery is limited. The MDEG framework is restricted to pairwise interactions, so that formalism was used under the assumption of a sparse network. The MAC game problem that we shall study below will show how this type of restriction can be removed by using the ASG framework (instead of the MDEG framework). The model can be described as follows: Time is slotted. At any given time $t$, a mobile finds itself competing with $N_t$ other mobiles for the access to a channel. $N_t$ is assumed to have Poisson distribution with parameter $\lambda$. We shall formulate this as a sequential anonymous game as follows.

- **Individual state** A mobile has three possible states: $F$ (full), $AE$ (Almost Empty) and $E$ (Empty).
- **Actions** There are two actions: transmit at high power $H$ or low power $L$. At state $AE$ a mobile cannot transmit at high power, while at $E$ it cannot transmit at all.
- **Transition probabilities** From state $AE$ the mobile moves to state $E$ with probability $p_E$ and otherwise remains in $AE$. At state $E$ the mobile has to recharge. It moves to state $F$ after one time unit. A mobile in state $F$ transmitting with power $r$ moves to state $AE$ with probability proportional to $r$ and given by $\alpha r$ for some constant $\alpha >0$.
- **Payoff** Consider a given cellular phone that transmits a packet. Assume that $x$ other packets are transmitted with high power and $y$ with low power to the same base station. A packet transmitted with low power is received successfully with some probability $q$, if it is the only packet transmitted, i.e., $y=0$, $x=0$. Otherwise it is lost. A packet transmitted with high power is received successfully with some probability $Q>q$, if it is the only packet transmitted at high power, i.e., $x=0$. The immediate payoff is $1$, if the packet is successfully transmitted. It is otherwise zero. In addition, there is a constant cost $c>0$ for recharging the battery. Aggregate utility for a player is then computed as long-time average of the per-period payoffs.

Suppose $p$ is the fraction of population that transmits at high power in state $F$, and that $\mu _F$, $\mu _{AE}$, and $\mu _E$ are fractions of players in respective states. Then probability of success for a player transmitting at high power is

$$\begin{aligned} QP(x=0)=Q(e^{-\lambda }+\sum _{k=1}^\infty \frac{\lambda ^k}{k!}e^{-\lambda }(1-p\mu _F)^k=Qe^{-\lambda }e^{\lambda (1-p\mu _F)}=Qe^{-\lambda p\mu _F}, \end{aligned}$$

while probability of success when a player transmits at low power is

$$\begin{aligned} qP(x+y=0)=q(e^{-\lambda }+\sum _{k=1}^\infty \frac{\lambda ^k}{k!}e^{-\lambda }\mu _E^k)=qe^{-\lambda }e^{\lambda \mu _E}=qe^{\lambda (\mu _E-1)}. \end{aligned}$$

These values do not depend on actual numbers of players applying respective strategies—only on fractions of players in each of the states using different actions. Thus, instead of considering an $n$-player game for any fixed $n$, it is reasonable to apply the anonymous game formulation with $\tau =[\tau _{F,H},\tau _{F,L},\tau _{AE,L},\tau _E]$ denoting the vector of fractions of players in respective states and using respective actions, with immediate rewards

$$\begin{aligned} u(s,a,\tau )=\left\{ \begin{array}{ll} Qe^{-\lambda \tau _{F,H}},&{} \text{ when } a=H,\\ qe^{\lambda (\tau _E-1)},&{} \text{ when } a=L,\\ -c,&{} \text{ when } s=E,\end{array}\right. \end{aligned}$$

and transition probabilities defined by matrix

$$\begin{aligned} \mathbb {Q}(a,\tau )=\left[ \begin{array}{lll} 1-\alpha a&{}\alpha a&{}0\\ 0&{}1-p_E&{}p_E\\ 1&{}0&{}0 \end{array}\right] . \end{aligned}$$

### The Solution

Stationary state of the chain of private states of a player using policy $f$ prescribing him to use high power with probability $p$ when in state $F$ is

$$\begin{aligned} \frac{1}{\alpha (pH+(1-p)L)(p_E+1)+p_E}\left[ p_E,\alpha (pH+(1-p)L),p_E\alpha (pH+(1-p)L)\right] . \end{aligned}$$

Thus, it can be computed that his expected average reward is of the form

$$\begin{aligned}&\frac{Ap+B}{Cp+D}\quad \text{ with }\\&A=p_EQe^{-\lambda \tau _{F,H}}+((H-L)\alpha -p_E)qe^{\lambda (\tau _{E}-1)}-c\alpha p_E(H-L),\\&B=(L\alpha +p_E)qe^{\lambda (\tau _E-1)}-c\alpha p_EL,\\&C=\alpha (H-L)(p_E+1),\quad D=\alpha L(p_e+1)+p_E. \end{aligned}$$

It can be either a strictly increasing, a constant or a strictly decreasing function of $p$, depending on whether $AD>BC$, $AD=BC$, or $AD<BC$, and thus the best response of a player against the aggregated state-action vector $\tau$ is $p=1$ when $AD>BC$, any $p\in [0,1]$ when $AD=BC$ or $p=0$ when $AD<BC$. This leads to the following conclusion: since by Theorem 3.1 this anonymous game has an equilibrium, one of the three following cases must hold:

- If
  $$\begin{aligned}&\left[ p_EQe^{-\frac{\lambda p_E}{\alpha H(p_E+1)+p_E}}+((H-L)\alpha -p_E)qe^{-\frac{\lambda (\alpha H+p_E)}{\alpha H(p_E+1)+p_E}}-a\alpha p_E(H-L)\right] \\&\quad \times \left[ \alpha L(p_E+1)+p_E\right] >\left[ (L\alpha +p_E)qe^{-\frac{\lambda (\alpha H+p_E)}{\alpha H(p_E+1)+p_E}}-c\alpha Lp_E\right] \\&\quad \times \left[ \alpha (H-L)(p_E+1)\right] , \end{aligned}$$
  then all the players use high power in state $F$ at equilibrium.
- If
  $$\begin{aligned}&\left[ p_EQe^{-\frac{\lambda p_E}{\alpha L(p_E+1)+p_E}}+((H-L)\alpha -p_E)qe^{-\frac{\lambda (\alpha L+p_E)}{\alpha L(p_E+1)+p_E}}-a\alpha p_E(H-L)\right] \\&\quad \times \left[ \alpha L(p_E+1)+p_E\right] <\left[ (L\alpha +p_E)qe^{-\frac{\lambda (\alpha L+p_E)}{\alpha L(p_E+1)+p_E}}-c\alpha Lp_E\right] \\&\quad \times \left[ \alpha (H-L)(p_E+1)\right] , \end{aligned}$$
  then all the players use low power in state $F$ at equilibrium.
- If none of the above inequalities holds, than we need to find $p^*$ satisfying
  $$\begin{aligned}&\left[ p_EQe^{-\lambda \tau _{F,H}}+((H-L)\alpha -p_E)qe^{\lambda (\tau _{E}-1)}-c\alpha p_E(H-L)\right] \\&\quad \times \left[ \alpha L(p_e+1)+p_E\right] \!=\!\left[ (L\alpha +p_E)qe^{\lambda (\tau _E-1)}-c\alpha p_EL\right] \left[ \alpha (H-L)(p_E+1)\right] \end{aligned}$$
  with $\tau _{F,H}=\frac{p^*p_E}{\alpha (p^*H+(1-p^*)L)(p_E+1)+p_E}$ and $\tau _E=\frac{\alpha (p^*H+(1-p^*)L)p_E}{\alpha (p^*H+(1-p^*)L)(p_E+1)+p_E}$. Then all the players use policy prescribing to use high power with probability $p^*$ in state $F$ at equilibrium.

#### *Remark 6.1*

It is worth noting here that some generalizations of the model presented above can be considered. We can assume that there are more energy levels and more powers at which players could transmit in our game (similarly as in [27]). We can also assume that the players do not always transmit, only with some positive probability (then the individual state becomes two-dimensional, consisting of player’s energy state and an indicator of whether he has something to transmit or not). Both these generalizations are tractable within our framework, though the computations become more involved.

## The Case of Linear Utility: Maintenance-Repair Example

### General Linear Framework

In the next example we consider a game satisfying some additional assumptions. Let $K=(S \times A )$. Let $\mathbf{u} (\tau )$ be a column vector whose entries are $u(k,\tau )$. We consider now the special case that $u(k,\tau )$ is linear in $\tau$.

Equivalently, there are some vector ${ \mathbf u^1 }$ over $K$ and a matrix $\mathbf{u^2}$ of dimension $|K| \times |K|$ such that

$$\begin{aligned} \mathbf{u} (\tau ) = \mathbf{u}^1 + \mathbf{u}^2 \tau . \end{aligned}$$

Similarly, we assume that the transition probabilities are linear in $\tau$. Then the game becomes equivalent to solving a symmetric bilinear game that of finding a fixed point of (2)–(3). Linear complementarity formulation can be used and solved using Lemke’s algorithm. From the solution $\tau$, the equilibrium $(f,\tau _S)$ can be derived with a help of equation (4).

### Maintenance-Repair Game: The Model

The maintenance-repair example presented below can be seen as a toy model. Its main purpose, however, is to show, how the abovementioned method can be used in a concrete game satisfying the linearity conditions mentioned above.

Each car among a large number of cars is supposed to drive one unit of distance per day. A car is in one of the **individual states:** good ($g$) or bad ($b$). When a car is in a bad state, then it has to go through some maintenance and repair actions and cannot drive for some (geometrically distributed) time.

A single driver is assumed to be infinitesimally “small” in the sense that its contribution to the congestion experienced by other cars is negligible.

We assume that there are two types of behavior of drivers. Those that drive gently, and those that take risks and drive fast. This choice is modeled mathematically through **two actions:** aggressive ($\alpha$) and gentle ($\gamma$). An aggressive driver is assumed to drive $\beta$ times faster than a gentle driver.

**Utilities** A car that goes $\beta$ times faster than another car, traverses the unit of distance at a time that is $\beta$ times shorter. Thus the average daily delay it experiences is $\beta$ times shorter. We assume that at a day during which a car drives fast, it spends 1/$\beta$ of the time that the others do. It is then reasonable to assume that the contribution to the total congestion is $\beta$ times lower than that of the other drivers. More formally, let $\eta$ be a delay function. Then the daily congestion cost $D$ of a driver is given as

$$\begin{aligned} u( g,\alpha ,\tau ) = u(g,\gamma , \tau )/\beta ,\\ u(g,\gamma , \tau ) = - \eta \left( \tau (g,\gamma ) + \tau (g , \alpha )/\beta ) \right) . \end{aligned}$$

For the state $b$ we set simply

$$\begin{aligned} u(b,a,\tau )= - 1, \end{aligned}$$

which represents a penalty for being in a non-operational state. It does not depend on $a$ nor $\tau$.

**Transition probabilities:** We assume that transitions from $g$ to $b$ occur due to collisions between cars. Further, we assume that the collision intensity between a car that drives at state $g$ and uses action $a$ is linear in $\tau$. More precisely,

$$\begin{aligned} Q( b | g , a , \tau ) = c_a^{\gamma } \tau (g,\gamma ) + c_a^{\alpha } \tau ( g , \alpha ) . \end{aligned}$$

We naturally assume that $c_\alpha ^a > c_\gamma ^a$ for $a=\alpha ,\gamma$ and that $c_a^\alpha > c_a^\gamma$ for $a=\gamma ,\alpha$. If a driver is more aggressive than another one, or if the rest of the population is more aggressive, then the probability of a transition from $g$ to $b$ increases. We rewrite the above as

$$\begin{aligned} Q( b | g , a , \tau ) = c_a \cdot \tau (g, . ) . \end{aligned}$$

If a randomized stationary policy is used which chooses $( \alpha , \gamma )$ with respective probabilities $( p_\alpha , p_{\gamma } ) =: \mathbf{p }$, then the one step transition from $Q$ to $b$ occurs with probability

$$\begin{aligned} Q( b | g , \mathbf{p} , \tau ) = \sum _{a = \gamma , \alpha } p_a c_a \cdot \tau (g, . ) =: \mathbf{p } \cdot c \cdot \tau (g , . ) . \end{aligned}$$

Once in state $b$, the time to get fixed does not depend any more on the environment, and the drivers do not take any action at that state. Thus $\psi := Q( g | b , a , \tau )$ is some constant that is the same for all $a$ and $\tau$.

### The Solution

We shall assume throughout that the congestion function $\eta$ is linear. It then follows that this problem falls into the category of Sect. 7.1.

Let $\tau$ be given. Let a driver use a stationary policy $\mathbf{p}$. Then the expected time it remains in state $g$ is

$$\begin{aligned} \sigma (\mathbf{p} ,\tau ) = \frac{1}{Q( b | g , \mathbf{p} , \tau ) }. \end{aligned}$$

Its total expected utility during that time is

$$\begin{aligned} W_g( \mathbf{p }, \tau ) = \sigma (\mathbf{p} ,\tau ) \sum _a p_a u(g,a,\tau ) = \sigma (\mathbf{p} ,\tau ) \sum _a p_a u(g,a,\tau ).\\ = - \sigma (\mathbf{p} ,\tau ) \left( p_\gamma + \frac{ p_\alpha }{ \beta } \right) \eta \left( \tau (g,\gamma ) + \tau (g , \alpha )/\beta ) \right) . \end{aligned}$$

The expected repair time of a car (the period that consists of consecutive time it is in state $b$) is given by $\psi ^{-1}$. Thus the total expected utility during that time is

$$\begin{aligned} W_b( \mathbf{p }, \tau ) = - \psi ^{-1}. \end{aligned}$$

Thus the average utility is given by

$$\begin{aligned} J(\mu , \mathbf{p } , \pi ( \tau ) ) = \frac{ W_g( \mathbf{p }, \tau ) \psi - 1 }{ \frac{\psi }{ Q( b | g , \mathbf{p} , \tau ) } + 1 }, \end{aligned}$$

where $\mu$ is an arbitrary initial distribution and where $\pi (\tau )$ is the stationary policy that is obtained from $\tau$ as in (4).

Let $\mathbf{p^* }$ be a stationary equilibrium policy and assume that it is not on the boundary, i.e., $0 < p_\alpha ^* < 1$. We shall consider the equivalent bilinear game. Let $\rho ^*$ be the occupation measure corresponding to $\mathbf{p}^*$. It is an equilibrium in the bilinear game.

Since the objective function is linear in $\rho$, $\rho ^*$ should be such that each individual player is indifferent between any stationary policy. In particular, we should have $J(\mu , 1_\alpha , \pi ( \tau ) ) = J(\mu , 1_\gamma , \pi ( \tau ) )$, where $1_a$ is the stationary pure policy that chooses always $a$.

We thus obtain the equilibrium occupation measure $\rho ^*$ as a $\tau$ that satisfies

$$\begin{aligned} \frac{ W_g( 1_\alpha , \tau ) \psi - 1 }{ \frac{ \psi }{ Q( b | g , \alpha , \tau ) } + 1 } = \frac{ W_g( 1_\gamma , \tau ) \psi - 1 }{ \frac{ \psi }{ Q( b | g , \gamma , \tau ) } + 1 }. \end{aligned}$$

The equilibrium policy $\mathbf{p^* }$ is obtained from $\rho ^*$ as in (4), and the equilibrium stationary measure is $\rho ^*_S$.

## Perspectives

The framework analyzed in this paper generalizes that of Jovanovic and Rosenthal [7], addressing the cases of expected average reward and total expected reward, which have not yet been studied in the literature. Lack of this kind of results so far is not so unexpected, if we take into account that game-theoretic tools were for many decades used mostly in economic contexts, where discounted rewards are usually more appropriate in multistage models. The time scales that are of interest in dynamic games applied to Engineering, and in particular to telecommunication networks, are often much faster than those occurring in economic models, which makes reward criteria focusing on the long-run behavior of the analyzed systems more appropriate. These are young and quickly developing fields of application of game theory that generate those new models.

Our paper tries to fill in the existing gap. It has to be noticed, however, that a lot remains to be done. First of all, the results presented here are limited to the games with finite state and action spaces. One natural generalization would be finding conditions for equilibria to exist in analogous games played on infinite, and possibly noncompact, state, and action sets. Second interesting problem is, whether similar results hold, when the global state of the game does not converge to some stationary distribution, at least under some subclass of stationary policies. In such a case not only the proofs may become more involved, but also the notion of equilibrium needs redefining. Finally, for applications it may be quite important to extend the present model to semi-Markov case, where the moments when a player makes his decisions remain discrete but follow some controlled impulse process.

Another important issue, addressed just briefly in Sect. 7.1, concerns designing tools for computing equilibria in such games. As it is widely known, one of the main problems when dealing with dynamic games, stochastic games in particular, when the number of players becomes large, is the so-called *curse of dimensionality*–in stochastic games the sizes of state and action spaces growing exponentially being a particular problem. Although equilibrium-existence results often exist for games with big finite number of players, the computation becomes impossible usually already for a relatively small number. The anonymous game formulation simplifies visibly the structure of the game and thus gives much bigger chances of computing the equilibria, which then could be used as approximate equilibria for the models with large finite number of players. In addition, the equilibria computed for the limiting, anonymous case, may have (under appropriate conditions) a property, used in Medium Access Game example presented in Sect. 6 that they are equally good approximations of equilibria of games with a different large number of players, which can be used when the exact number of players is unknown. In Sect. 7.1 we present a way to transform the problem of finding equilibrium in an anonymous game with linear utilities into that of finding an equilibrium in a bimatrix game. Designing similar tools for more general case is another important problem to solve.

Finally, the resemblance of the framework considered in this paper to that of the classical traffic assignment problem suggests, it may be natural to try to extend tools from the traffic assignment framework to the one studied in this paper, such as the convergence of dynamic decentralized learning schemes (e.g., the replicator dynamics). More generally, it remains to study, whether anonymous sequential games provide good approximations for similar games but with finitely many players, and vice versa. Our paper answers this question only in a very limited range, while this type of question is frequently asked in mean field games, which are games played by an infinite population of players, see [40] and references therein.

## Conclusions

The framework of the game defined in this paper is similar in nature to the classical traffic assignment problem in that it has an infinity of players. In both frameworks, players can be in different states. In the classical traffic assignment problem, a class can be characterized by a source-destination pair, or by a vehicle type (car, pedestrian or bicycle). In contrast to the traffic assignment problem, the class of a player in our setting can change in time. Transition probabilities that govern this change may depend not only on the individual’s state but also on the fraction of players that are in each individual state and that use different actions. Furthermore, these transitions are controlled by the player. A strategy of a player of a given class in the classical traffic assignment problem can be identified as the probability it would choose a given action (path) among those available to its class (or its “state”). The definition of a strategy in our case is similar, except that now the probability for choosing different actions should be specified not just in one state.

Our paper provides definitions and tools for the study of anonymous sequential games with the total cost and with the average cost criteria, which have not been covered in the existing literature. It provides conditions for the existence of stationary equilibrium, and illustrates through several examples, how to compute it. The contribution of the paper is not only in extending previous results to new cost criteria but also in providing an appropriate definition of equilibria for the new cost criteria.

## Footnotes

1 Here and in the sequel for any set $B$, $\Delta (B)$ denotes the set of all the finite-support probability measures on $B$. In particular, if $B$ is a finite set, it denotes the set of all the probability measures over $B$. In such a case we always assume that $\Delta (B)$ is endowed with Euclidean topology.

2 Note that its transition is deterministic.

3 Note that we assume this stationary policy may depend on $\tau$, as we consider the properties of the Markov chain of individual states of a player under fixed state-action distribution of all the other players.

4 All the properties appearing in the assumptions are commonly used in the Markov decision processes literature. Those readers who are not familiar with them or are interested in mutual relationships between these properties are referred to [31, 33].

5 The example is a reworking of Example 3. in [34].

6 Of course a stationary policy is only a function of individual state $s$ here.

7 Here and in the sequel $\delta [x]$ denotes a probability measure concentrated in $x$.

8 Here it does not depend on $\tau$ by (AT1).

9 Note that a measure over the set of states of an aperiodic Markov process can be invariant, only if it is a convex combination of unique invariant measures on each of the ergodic classes. The coefficients of this convex combination are exactly the $\mu ^*_l$.
